# New pathogenic insights from large animal models of neurodegenerative diseases

**DOI:** 10.1007/s13238-022-00912-8

**Published:** 2022-03-25

**Authors:** Peng Yin, Shihua Li, Xiao-Jiang Li, Weili Yang

**Affiliations:** Guangdong Key Laboratory of Non-human Primate Research, Guangdong-Hongkong-Macau Institute of CNS Regeneration, Jinan University, Guangzhou, 510632 China

**Keywords:** large animal models, neurodegenerative diseases, CRISPR/Cas9

## Abstract

Animal models are essential for investigating the pathogenesis and developing the treatment of human diseases. Identification of genetic mutations responsible for neurodegenerative diseases has enabled the creation of a large number of small animal models that mimic genetic defects found in the affected individuals. Of the current animal models, rodents with genetic modifications are the most commonly used animal models and provided important insights into pathogenesis. However, most of genetically modified rodent models lack overt neurodegeneration, imposing challenges and obstacles in utilizing them to rigorously test the therapeutic effects on neurodegeneration. Recent studies that used CRISPR/Cas9-targeted large animal (pigs and monkeys) have uncovered important pathological events that resemble neurodegeneration in the patient’s brain but could not be produced in small animal models. Here we highlight the unique nature of large animals to model neurodegenerative diseases as well as the limitations and challenges in establishing large animal models of neurodegenerative diseases, with focus on Huntington disease, Amyotrophic lateral sclerosis, and Parkinson diseases. We also discuss how to use the important pathogenic insights from large animal models to make rodent models more capable of recapitulating important pathological features of neurodegenerative diseases.

## Introduction

The prevalence of age-dependent neurodegenerative diseases (NDs), such as Alzheimer’s disease (AD), Parkinson’s diseases (PD), Amyotrophic lateral sclerosis (ALS), and Huntington’s disease (HD), has increased worldwide in parallel with the rise in life expectancy. The common features of these diseases include selective neurodegeneration of specific populations of neurons that occurs in an age-dependent manner in the human brain (Wyss-Coray, 2016). The selective neurodegeneration in NDs is closely associated with the accumulation of misfolded proteins in the affected brain regions, which is largely due to the impairment of cellular machinery to clear unwanted and toxic proteins or peptides in aged neurons (Rubinsztein, 2006). Thus, identification of a treatment of any of these NDs would have broad implications for developing therapeutics for a large number of patients with NDs.

So far, there has been no effective treatment of NDs despite great efforts and progress being made over the course of several decades. The failure to find effective cues for NDs is largely owing to our incomplete understanding of the pathological mechanism underlying selective neurodegeneration. Most of NDs cases are sporadic with unknown causes, making it difficult to investigate the pathogenesis of these diseases. However, some NDs are caused by genetic mutations, which have facilitated research on investigation of how the mutations cause the disease proteins to become misfolded and toxic to initiate selective neurodegeneration. AD, which is characterized by dementia resulting from extensive yet selective neuron death in the neocortex and hippocampus, is the most common NDs with more than 90% of patients being sporadic and less than 10% of AD cases caused by a single genetic mutation in the *APP* genes (Presenilin 1, Presenilin 2, and Amyloid precursor protein APP) (Lanoiselée et al., 2017). PD is the second most common NDs that affects more than 1% people over age 60. The pathologic hallmarks of PD are the preferential loss of dopamine (DA) neurons and formation of Lewy body inclusions in the substantia nigra pars compacta (Damier et al., 1999). Although the majority of PD cases are sporadic, approximate 10% PD patients are caused by mutations in the genes for alpha-synuclein, PINK1, Parkin, LRRK2 and other proteins (Deng et al., 2018). ALS is also a progressive neurodegenerative disease that particularly affects motor neurons in the brain and the spinal cord, resulting in the loss of muscle movement (Grad et al., 2017). About 5%–10% of ALS patients are the familial form of ALS that could be caused by various mutations of genetic loci, including TAR DNA-binding protein 43 (*TDP-43*), superoxide dismutase 1 (*SOD1*), fused in sarcoma (*FUS*), and *C9ORF72* (Neumann et al., 2006; Turner et al., 2013). Of many important NDs, HD is a mono-genetic disorder and represents a family of NDs that are caused by a CAG repeat expansion, which is translated to an expanded polyglutamine (polyQ) repeat in the disease proteins (Orr and Zoghbi, 2007; Tabrizi et al., 2020). In HD, the polyQ expansion encoded by an expanded CAG repeat (>36 CAGs) in exon 1 of the HD gene causes huntingtin (HTT) to misfold and aggregate in the patient brain, resulting in the preferential loss of the medium spiny neurons in the striatum and extended neurodegeneration in various brain regions as HD progresses (Bates et al., 2015). The genetic mutations identified in NDs have enabled the generation of various animal models harboring the disease mutations, which have greatly advanced our understanding of the pathogenesis of NDs.

Animal models that can recapitulate key pathological changes in the patient brains would be important for developing effective therapeutic strategies. Various species of animal models for NDs have been investigated, including fruit flies, rodents, dogs, sheep, pigs and non-human primates (Dawson et al., 2018). Among those species, rodents are the most widely used animal model for generating disease models due to their relatively short life span, easier and rapid breeding, lower costs, and available genetic editing techniques. Particularly, there are versatile genetic tools to establish loss- or gain-of-function mouse models, and genetically modified rodent models have provided important insights into the pathogenesis and therapeutics for neurodegenerative diseases. However, most of these rodent models fail to recapitulate the overt and typic neurodegeneration seen in the patients (Deng and Siddique, 2000; Crook and Housman, 2011; Dawson et al., 2018). This drawback apparently prevents the rigorous evaluation of the therapeutic effects on neurodegeneration. Recently, several large animal models carrying human genetic mutations are found to more faithfully mimic the important nature of selective neurodegeneration (Yang et al., 2021a). Because large animal models of HD, PD, and ALS have been successfully established and published, we will focus on the animal models of these diseases and discuss the important pathogenic insights from the large animal models.

## Lack of overt neuronal loss in rodent models of neurodegenerative diseases

It would be worth discussing some important rodent models of NDs prior to describing large animal models, as these rodent models have been widely studied in the field and shed light on the pathogenesis of NDs. Various genetically modified mouse models of HD have been established and revealed that N-terminal fragments of mutant Htt with expanded polyQ repeats can accumulate in the brain to affect movement and neuronal function (Crook and Housman, 2011; Farshim and Bates, 2018). This idea is supported by the more severe phenotypes of transgenic mice expressing truncated N-terminal Htt fragments than those caused by full-length mutant Htt (Farshim and Bates, 2018). Because overexpressing N-terminal mutant Htt could cause phenotypes that may not occur in humans when full-length mutant Htt is expressed at the endogenous level, several HD knock-in mouse models were generated to express an expanded CAG/glutamine repeat in the endogenous mouse HD gene. However, there is no overt and selective medium spiny neuronal loss in HD knock in mouse models (Levine et al., 2004; Crook and Housman, 2011).

Mutations in the human *PINK1* and *PRKN* gene were found to result in autosomal recessive Parkinson’s disease (PD) that is featured by neurodegeneration in association with mitochondria dysfunction (Valente et al., 2004; McInerney-Leo et al., 2005; Corti et al., 2011; Pickrell and Youle, 2015). However, the *Pink1* or *Prkn* KO mouse models are unable to recapitulate selective and overt neurodegeneration seen in PD (Kitada et al., 2007; Xiong et al., 2009; Gispert et al., 2009; Akundi et al., 2011) or validate the important in vitro findings for the function of PINK1/Parkin in mitophagy (Whitworth and Pallanck, 2017; Cummins and Gotz, 2018). Moreover, triple knockout of three PD related genes of *Prkn*/*Pink1*/*DJ-1* at the same time in mouse models have not shown any obvious neuronal degeneration even in very old age (Kitada et al., 2009).

Since mutations in the nuclear *TDP-43* gene cause ALS, rodent models carrying *TDP-43* mutations were widely used to investigate ALS pathogenesis. TDP-43 is a nuclear protein that is involved in a variety of cellular functions including gene transcription, RNA processing, and protein homeostasis (Lagier-Tourenne and Cleveland, 2009; Polymenidou et al., 2011; Weishaupt et al., 2016). In patients with *TDP-43* mutations or some pathological conditions such as fronto-temporal lobar degeneration (FTLD), however, the nuclear TDP-43 is redistributed in the cytoplasm and forms cytoplasmic inclusions (Neumann et al., 2006; Arai et al., 2006; Chen-Plotkin et al., 2010). This cytoplasmic redistribution of TDP-43 protein in human brains can lead to a loss of function in the nucleus and a gain of toxicity in the cytoplasm (Huang et al., 2012; Philips and Rothstein, 2015). Although some mouse models can have the minimal level of cytoplasmic TDP-43 (Wegorzewska et al., 2009; Wils et al., 2010; Mitchell et al., 2015), most of *TDP-43* mutant mice show the predominantly nuclear localization of TDP-43 and do not reproduce the key pathological hallmark of cytoplasmic mislocalization of TDP-43 (Wegorzewska et al., 2009; Shan et al., 2010; Huang et al., 2012; Yan et al., 2014; Philips and Rothstein, 2015).

Thus, although the mouse models can remarkably recapitulate protein misfolding and aggregation seen in the patient brains (Zoghbi and Botas, 2002; Dugger et al., 2017; Soto and Pritzkow, 2018), most of the mouse models cannot fully mimic the symptoms and pathologies of these neurodegenerative diseases. This phenomenon could be due to species differences determined by genomic, molecular, and anatomic differences between rodents and humans.

## Pronounced differences in the brains of different species

**Figure 1:**
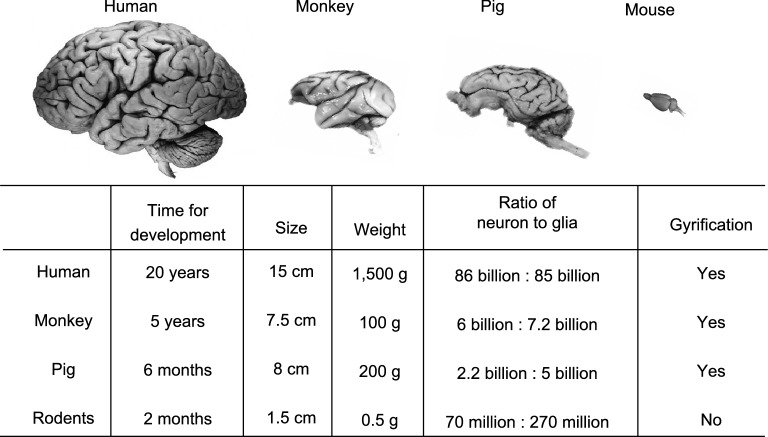
**Major differences in brain size and structures between rodents and large animals**

There are considerable differences in brain development, anatomical structure, cognitive and behavioral complexity between small and large animals, which explain why many human neurological and neuropsychiatric diseases are inadequately modeled in rodents (Izpisua Belmonte et al., 2015). For example, a larger cortical progenitor pool in the outer subventricular zone (SVZ), which is not present in rodents (Smart et al., 2002), is required for cortical gyrification (Rash et al., 2019). Otani et al. described that cortical progenitor cells expand their population for an extended period during the neurogenesis in primates (Otani et al., 2016), but such expansion is not prominent in rodents (Gao et al., 2014). Consequently, the cerebral cortex differs 1000-fold in size between mouse and large animals (Defelipe, 2011; Herculano-Houzel, 2012). As a result, rodent brains are much smaller and lack the folding of the cortical surface whereas pig, monkey, and human share this important feature in their brains (Fig. 1). The increased neuronal number and cerebral cortex size lead to more complex neural architectures that determine cognitive abilities (Herculano-Houzel, 2012; Geschwind and Rakic, 2013) and can also impact pathological and behavioral phenotypes of animal models of NDs.

Aging processes also differ significantly among different types of animals. Rodents normally live for less than 3 years, a short life span that may not be sufficient for the occurrence of neurodegeneration that usually takes decades to happen in humans. The rapid development and accelerated aging in small animals could render their brains or neuronal cells less vulnerable to insults or toxic proteins such that they lack robust neurodegeneration despite the presence of impaired brain function.

At the molecular level, the brains of large animals and humans share more similar patterns of gene expression than does the mouse brain (Bernard et al., 2012; Zeng et al., 2012; Hawrylycz et al., 2015; Bakken et al., 2016; Sousa et al., 2017; Zhu et al., 2018). Especially, transcriptional profiling in adult mice and humans demonstrated robust differences between neuronal and non-neuronal cell types and large-scale changes over the course of development (Darmanis et al., 2015; Molyneaux et al., 2015; Zeisel et al., 2015). In rat and human, there are 22% of genes showing different developmental trajectories, whereas only 9% of genes have different trajectories between rhesus monkey and human (Bakken et al., 2016). Thus, the brains of large animals may express the same molecules that are important for neuronal survival or death in humans but may not be essential in rodents or small animals.

Although pig is less similar to human than monkey, especially in cognition and emotion, the global structure of the pig brain is approximately equal to that of humans. In addition, the body size and physiology of pigs resemble those of humans, allowing for safe dosage ranges to be defined in drug development studies and toxicological testing. Pigs have an early sexual maturity (5–8 months) as well as a relatively short gestation period (about 114 days) and are able to produce multiple offspring (about 10–12 piglets per liter). In addition, the pig endogenous genes can be edited via somatic cell nuclear transfer (SCNT) to produce knock-in or knockout models. All these advantages make pigs a promising and alternative large animal model for investigating human diseases (Lunney et al., 2021).

## Strategies for generating large animal models of neurodegenerative diseases

Identification of genetic mutations responsible for NDs opened up an avenue to genetically express the same mutant genes in animals to model NDs. Unlike mouse models that can be generated using their embryonic stem cells after *in vitro* genome editing, large animal models were often generated by transgenic approach that introduces exogenous mutant genes in the fertilized eggs. However, the phenotypes of transgenic animals are influenced by the copy numbers of the transgene and its location in the host genome. The recently developed new genome-editing technologies, such as the CRISPR/Cas9 system, makes revolutionary changes in modifying endogenous genomes in large animals. For example, germline genome editing in non-human primates can be achieved by injection of the CRISPR/Cas9 system into the fertilized eggs (Zhao et al., 2017; Zhang et al., 2018; Yang et al., 2019b; Zhou et al., 2019). Since most genetic diseases result from point mutations, the development of base editor system is particularly useful to introduce or correct a point mutation in the endogenous genes (Xie et al., 2019; Chen et al., 2020; Wang et al., 2020).

However, the long-life cycle and high costs of non-human primates prevent the widespread use of genetically modified monkey models. Since CRISPR/Cas9 can also target genes in adult neuronal cells (Incontro et al., 2014; Swiech et al., 2015; Yang et al., 2021b), it can be directly applied to the brains of adult monkeys via stereotaxic injection of viral expression vectors. Such studies would allow one to explore the function of mutant genes in adult monkey and also to more rapidly generate monkey models that can mimic brain region-specific or age-dependent neurodegeneration. For example, because of the gain of toxicity of α-synuclein, stereotaxic injection of lentiviral vectors expressing mutant α-synuclein (A53T) into the substantia nigra of monkeys at different ages was performed (Yang et al., 2015). Li et al. also used AAV9-delivered CRISPR/Cas9 system to directly co-edit *PINK1* and *DJ-1* genes in the substantia nigras (SNs) of monkeys at different ages (Li et al., 2021). Our group recently depleted the monkey brain *PINK1* gene via AAV-mediated CRISPR/Cas9 in adult monkey brain (Yang et al., 2021b). Furthermore, genome editing could be used to modify genes in different cell types (such as neurons or glia) with specific promoters for investigating gene functions in different brain cell types.

**Figure 2:**
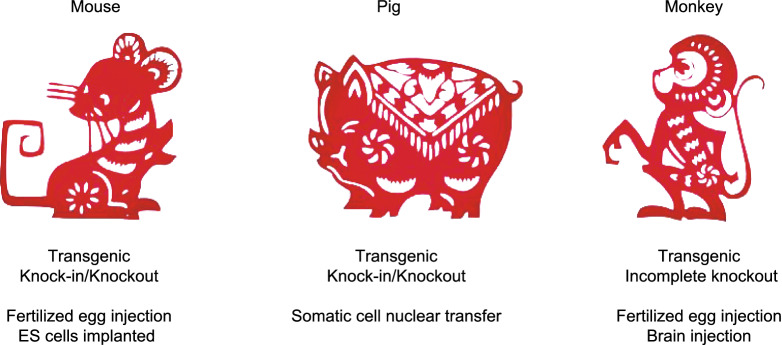
**Common strategies to generate animal models of neurodegenerative diseases**

Pig models of NDs can be established using SCNT to introduce genetic mutations in the endogenous pig genes to generate knock-in and knockout models (Hauschild et al., 2011; Zhou et al., 2015; Ryu et al., 2018; Yan et al., 2018). Wang et al. established a pROSA26-Cas9 pig line with Cre-inducible Cas9 expression to facilitate genome editing in pigs (Wang et al., 2017). Expression of the mutant genes at the endogenous level would avoid artificial effects of overexpressed transgene. Furthermore, SCNT in combination with CRISPR/Cas9 allowed for selecting embryos expressing a single mutation, leading to the expression of the same mutant gene ubiquitously in each cell and eliminating the concern of mosaicism of gene targeting caused by CRIPSR/Cas9. The development of genome editing technology has accelerated the use of pigs, non-human primates, and other large animals to model human diseases (Fig. 2).

## Genetically modified large animal models of neurodegenerative diseases

### Huntington’s disease (HD)

Genetically modified large animal models are summarized in Table 1. Of these animal models, HD transgenic monkeys were the first transgenic monkey model of human diseases, which was established by injecting lentiviruses into fertilized rhesus monkey oocytes to express exon 1 (1–67) mutant HTT containing 84Q (Yang et al., 2008). The HD monkeys showed abundant HTT aggregates in the neuronal nuclei and processes as seen in the brains of HD mouse models and patients, but died postnatally with severe neurodegeneration (Yang et al., 2008), in contrast to the transgenic HD mice that expressed the same transgenic *HTT* but could survive after birth without overt neurodegeneration (Davies et al., 1997).

**Table 1:** Large animal models of neurodegenerative diseases.

**Disease**	**Genetic anomaly**	**Species**	**Modifications Approach**	**Pathology and phenotypes**	**Reference**
**Huntington’s**	*HTT*	Pig	Embryonic expression of transgenic N-terminal mutant HTT (N548)	No neurodegeneration and gross motor deficits,	(Baxa et al., 2013)
	*HTT*	Pig	Embryonic knock-in of mutant *HTT*	Age-dependent neurological symptoms and neurodegeneration	(Yan et al., 2018)
	*HTT*	Monkey	Embryonic expression of transgenic exon 1 mutant *HTT*	Neurodegeneration and postnatal death	(Yang et al., 2008)
	*HTT*	Monkey	Brain specific expression of transgenic mutant *HTT*	Postnatal death, and clinical HD symptoms	(Weiss et al., 2020)
	*HTT*	Sheep	Embryonic expression of transgenic *HTT*	No report on obvious neurodegeneration, mild behavioral phenotypes	(Jacobsen et al., 2010; Reid et al., 2013)
**Parkinson’s**	*SNCA*	Monkey	Embryonic expression of transgenic: α-synuclein	Age-dependent non-motor symptoms and Lewy neurites	(Niu et al., 2015)
	*PINK1/PRKN/DJ-1*	Pig	Embryonic knockout of *PINK1*, *PRKN*, and *DJ-1*	No obvious neuronal loss, normal behavior	(Wang et al., 2016; Zhao et al., 2019)
	*PINK1/PRKN*	Pig	Embryonic knockout of *PINK1* and *PRKN*	No obvious neuronal loss, normal behavior	(Zhou et al., 2015)
	*PINK1*	Monkey	Embryonic targeting PINK1 by CRISPR/Cas9	Severe neuronal loss, Motor function deficits	(Yang et al., 2019b, a)
	*PINK1/DJ-1*	Monkey	Brain regional targeting *PINK1* and *DJ-1* by CRISPR/Cas9	Classic PD symptoms, severe nigral dopaminergic neuron loss	(Li et al., 2021)
	*PINK1*	Monkey	Brain regional targeting *PINK1* by CRISPR/Cas9	Severe neuronal loss Motor function deficits	(Yang et al., 2021b)
	*PINK1*	Monkey	Embryonic targeting *PINK1* by CRISPR/Cas9	No obvious neurodegeneration and phenotypes	(Chen et al., 2021)
**ALS**	*hSOD1(G93A)*	Pig	Embryonic expression of transgenic *SOD1*	Hind limb movement deficits, loss of motor neurons, formation of neuronal intranuclear inclusions in early disease stage	(Yang et al., 2014)
	*hSOD1(G93A)*	Pig	Embryonic expression of transgenic *SOD1*	No ALS-like phenotypes	(Chieppa et al., 2014)
	*TDP-43 (M337V)*	Pig	Embryonic expression of transgenic *TDP-43*	Severe phenotypes and early death.	(Wang et al., 2015)
	*TDP-43*	Monkey	Brain regional expression of transgenic *TDP-43*	Progressive motor weakness and muscle atrophy Cytoplasmic mislocalization of TDP-43	(Uchida et al., 2012)
	*TDP-43*	Monkey	Brain regional expression of transgenic *TDP-43*	Cytoplasmic accumulation of mutant TDP-43, motor function deficits	(Yin et al., 2019)

Different transgenic pigs were also generated to model HD. Transgenic pigs expressing N-terminal mutant HTT 105Q (N208-105Q) were generated via SCNT, but most of the transgenic HD piglets died postnatally (Yang et al., 2010). Another group generated transgenic HD pigs via lentiviral infection of porcine embryos that expressed a large fragment of mutant HTT (1–548) containing 124Q. However, these transgenic HD pigs showed much milder phenotypes and did not die after birth (Baxa et al. 2013; Schuldenzucker et al., 2017). Transgenic sheep that expressed full-length mutant HTT with 73Q and were also generated via microinjection into pronuclei of single-celled zygotes show very mild phenotypes (Jacobsen et al., 2010). Because viral infection or microinjection of fertilized oocytes can lead to various degrees of transgene expression in different types of cells, the distinct phenotypes in transgenic HD animals are clearly dependent on the transgene expression.

To express the mutant *HTT* gene in the endogenous genome of pigs, a HD knock-in pig model was generated via CRISPR/Cas9 and SCNT. This HD KI pig model expressed an expanded polyCAG (150 CAG) in the endogenous *HTT* gene (Yan et al., 2018). Importantly, when full-length mutant HTT with 150Q was endogenously expressed in this HD pig model, it caused striking and selective neurodegeneration as well as movement disorders, effectively recapitulating the typical pathological and clinic features in HD patients. Furthermore, the expanded CAG repeats and neurological phenotypes of these HD KI pigs can be transmitted to next generations by germline (Yan et al., 2018), providing a valuable model for investigating the pathogenesis and therapeutics of HD.

### Parkinson’s disease (PD)

Transgenic monkey models that overexpress mutant α-synuclein have been generated for investigating PD pathology and validated the neurotoxicity of mutant proteins (Giasson et al., 2002; Lee et al., 2002; Yang et al., 2015; Ip et al., 2017). However, the various expression levels of exogenous mutant proteins could compromise the phenotype outcomes such that it is difficult to compare the merits of each transgenic PD animal model. Based on the fact that genetic mutations in the *PINK1*, *PRKN* and *DJ-1* genes caused PD via a loss-of-function mechanism, CRISPR/Cas9-targeting of these genes in the pigs and monkeys was performed. Interestingly, disrupting the *PINK1* and *PRKN* genes in pigs via CRISPR/Cas9 did not produce any neurodegeneration and severe phenotypes (Zhou et al., 2015; Wang et al., 2016), like *Pink1* KO mouse models that show no neurodegeneration or severe phenotypes (Zhou et al., 2007; Gautier et al., 2008; Kitada et al., 2009). Chen et. al utilized the paired gRNA/Cas9-D10A nickases to disrupt the monkey *PINK1* in the fertilized monkey oocytes but did not observe PD phenotypes in the live mutant monkeys (Chen et al., 2021). It remains unknown whether the PINK1 protein is reduced in the brain of their monkey model, as only *PINK1* mRNA from the targeted fibroblasts was reported to be decreased. Because the mosaicism of CRISPR/Cas9-mediated mutations can induce different extents of PINK1 loss and varying degrees of phenotypes and because the deficiency of PINK1 protein is correlated with the loss of neuronal cells in different tissues in the same monkey (Yang et. al., 2019b), it is important to examine the protein level of the targeted gene in different brain regions. On the other hand, injection of AAV into the monkey substantia nigra to co-edit the *PINK1* and *DJ-1* genes could mediate severe nigra dopaminergic cell loss and motor function deficits (Li et al., 2021).

It seems likely that the phenotypes of *PINK1* targeted monkeys rely on the extent to which PINK1 is depleted. To ensure that PINK1 expression and function are completely lost, we used two gRNAs to disrupt the monkey *PINK1* exon 2 and exon 4, resulting in a large *PINK1* DNA fragment deletion in the monkey embryos. The newborn monkeys showed severe neurodegeneration or died postnatally (Yang et al., 2019b), demonstrating for the first time that PINK1 is essential for neuronal survive in the primate brain.

The phenotypes of *PINK1* targeted monkeys are clearly different from the rodent models with deletion of the mouse *Pink1* gene. None of *Pink1* knockout mouse models showed the typical degeneration of dopaminergic neurons seen in the brains of PD patients (Perez and Palmiter, 2005; Zhou et al., 2007; Kitada et al., 2007, 2009). Similarly, deletion of the gene for Parkin, which works together with Pink1 in protecting against mitochondrial damage, did not produce any obvious degeneration either in the mouse brain (Goldberg et al., 2003; Perez and Palmiter, 2005; Kitada et al., 2009). The remarkable differences between rodents and monkeys in *PINK1* deletion-mediated phenotypes underscore the value of using the non-human models to investigate the function of PINK1.

### Amyotrophic lateral sclerosis (ALS)

The absence of predominant distribution of mutant TDP-43 in the cytoplasm, a pathological hallmark of ALS, in the rodent models motivated us to use non-human primates for investigation. We previously created the *TDP-43* transgenic pig model that exhibited the cytoplasmic distribution of TDP-43 as seen in patient brains (Wang et al., 2015). To investigate the subcellular distribution of mutant TDP-43 in the monkey brain, we directly injected viral vector expressing mutant TDP-43 into the rhesus monkey brain cortex and substantia nigra (Yin et al., 2019). The majority of mutant TDP-43 was distributed in the cytoplasm of the monkey brain, contrary to the nuclear accumulation of TDP-43 in the mouse brain (Yin et al., 2019) but consistent with the cytoplasmic distribution of mutant TDP-43 in the monkey spinal cord (Uchida et al., 2012).

The human *SOD1* mutations could cause familial ALS. Although SOD aggregates are mainly found in the cytoplasm, intranuclear SOD1 aggregates were also seen in some postmortem brains of ALS patients with *SOD1* mutations (Kakita et al., 1997; Seilhean et al., 2004; Forsberg et al., 2011). Mutant *SOD1* transgenic mice are most widely used animal models of ALS and have given us important insights into the pathogenesis of ALS (Ferraiuolo et al., 2011; Philips and Rothstein, 2015). However, most of the transgenic *SOD1* mouse models did not show intranuclear aggregates and lack substantial cortical motor neuronal degeneration, which is a fundamental feature of ALS patients (Philips and Rothstein, 2015). Yang et al. generated the *SOD1* (*G*93A) transgenic pigs via the SCNT method (Yang et al., 2014). The transgenic *SOD1* pigs showed hind limb motor defects and neuronal degeneration in dose- and age-dependent manners. Also, in the early disease stage, mutant hSOD1 did not form cytoplasmic inclusions, but showed nuclear accumulation and ubiquitinated nuclear aggregates, as seen in some ALS patient brains (Yang et al., 2014). The differences between transgenic *SOD1* mice and pigs further support the idea that large animal models can more faithfully mimic the age-dependent and progressive pathological changes seen in the patient brains.

## New insights from large animal models into disease mechanisms

A successfully established animal model of neurodegenerative disease should adequately recapitulate the important pathological features and important clinical phenotypes of patients. The current large animal models reviewed above have demonstrated important species-dependent differences in neuropathology. They would serve as important tools to investigate the pathogenesis and pathological events that may uniquely occur in humans and can therefore be new therapeutic targets.

HD KI pigs can be used to rigorously evaluate the involvement of mutant HTT in a number of important pathological events found in small animal models. One of the well-documented events in HD mice is the somatic CAG repeat instability in the striatum, a brain region that is preferentially affected in HD (Swami et al., 2009). Further investigation of CAG repeat lengths in various brain regions of HD KI pigs showed that the CAG repeats are fairly stable in the striatum (Bai et al., 2021). These findings point to the potential regulation of the somatic instability of CAG repeats by species-dependent factors and stress the role of polyglutamine toxicity rather than CAG instability in large animals. The therapeutic implication of these findings is that removing mutant HTT protein would be more important than correcting CAG instability at the DNA level.

**Figure 3:**
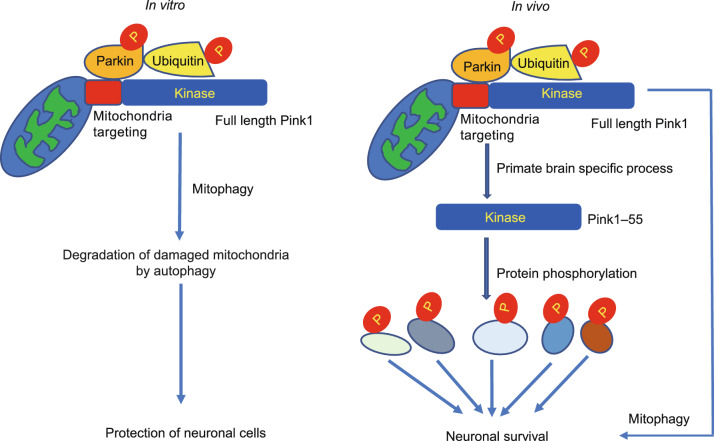
**New pathogenic insight of monkey models with *PINK1* mutation.** The prevalent theory for the function of PINK1 in mitophagy is largely based on *in vitro* studies (left). The *in vivo* studies of the primate brains suggest that PINK1 is a kinase to phosphorylate many neuronal proteins to maintain neuronal survival (right)

Comparison of *PINK1* KO models of mouse and monkey uncovered the unique expression and function of PINK1 in the primate brains, because PINK1 is undetectable in the mouse brain but abundantly expressed in the primate brain at the protein level (Yang et al., 2021b). This striking difference may explain why *Pink1* knock out mouse models do not have overt neurodegeneration because Pink1 is not essential in rodents. PINK1 seems to have mitochondria-independent function and can phosphorylate a series of neuronal function-related proteins that are vital to neuronal survival in the primate brains (Yang et al., 2019a; Sun et al., 2021). Thus, genetic mutations such as a point mutation or a mutation in the non-kinase domain of PINK1 created in other monkey models may not be able to significantly affect PINK1 kinase function to elicit obvious neurodegeneration (Chen et al., 2021). Characterization of *PINK1* targeted monkeys leads to a new theory that the primate PINK1 mainly functions as a kinase to maintain neuronal survival, which is different from the prevailing theory of mitophagy that is largely based on *in vitro* studies of stressed or damaged mitochondria (Fig. 3). The findings also implicate that improvement of dysfunction of PINK1 kinase, which is a druggable target, may be more effective than repair of damaged mitochondria in treating *PINK1* mutation-mediated pathology in PD.

**Figure 4:**
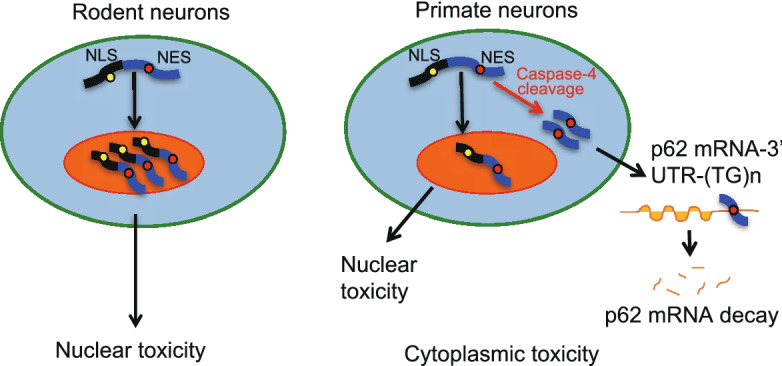
**New pathogenic insight of monkey models expressing mutant TDP-43.** TDP-43 remains in the nucleus of rodent neurons to elicit nuclear toxicity. In the primate neurons, however, the primate-specific caspase-4 cleaves TDP-43 to cause truncated TDP-43 to redistribute in the cytoplasm, resulting in the cytoplasmic toxicity

The distribution of mutant TDP-43 (M337V) in the cytoplasm in the brains of rhesus monkeys and pigs (Uchida et al., 2012; Wang et al., 2015; Yin et al., 2019) highlights the values of large animal models for investigating the cytoplasmic toxicity of TDP-43. Subsequent investigation identified that the primate-specific caspase-4, which is not expressed in the mouse brain, was able to cleave TDP-43 and remove its NLS-containing N-terminal domain, resulting in the cytoplasmic accumulation of TDP-43 (Yin et al., 2019). The cytoplasmic mutant TDP43 could selectively reduce SQSTM1 expression in the monkey brain by reducing *SQSTM1* mRNA stability via its binding to the unique 3’UTR sequence (GU/UG) of the primate *SQSTM1* transcript (Fig. 4) (Yin et al., 2021). Thus, selective expression of modifiers or targets of the disease proteins in large animal models apparently contributes to the specific neuropathological events that may not occur in small animal models.

## Challenges of using large animal models and resolutions

Although recently established large animal models of NDs offer fresh and important insights into the pathogenesis of NDs, there are notable limitations for generating large animal models. The high animal cost, the current low efficiency in gene targeting, especially for knock-in and conditional knockout or gene expression, in large animals and extensive time required for studies of large animals are obvious concerns for widely using such animal models for research. For example, pigs have a sexual maturity of 5–8 months and the gestation period of about 114 days. For monkeys, the sexual maturity takes much longer time (4–5 years), the gestation period lasts for about 165 days, and each female animal often produces a single offspring each year. In addition, generation of embryonic genome edited non-human primate models is technically challenging and labor intensive, as multiple processes including hormonal superovulation, embryo transfer with surgical implantation, and experienced animal care, would require the well-equipped animal facility, enough animal resources, and a highly trained team.

Additionally, CRISPR/Cas9 system creates the mosaic mutations that may result in various types of mutations and different cellular phenotypes. The mosaicism in offspring can be reduced by outcrossing the mosaic founders with wild type animals. However, it will take years to eliminate mosaicism in large animal models, especially for the non-human primates that require 4–5 years of sexual maturity to produce next generation. In pig models, however, the gene editing tools combined with SCNT have been successfully to establish the pig models that carry the same single genetic mutation as in human patients. SCNT was also applied to the non-human primate, and the results demonstrate the feasibility to generate knock-in non-human primate models (Liu et al., 2018), but the efficiency is much lower.

Several other technical hurdles also need to be resolved. Although the mosaic mutations in different animals may not yield the same phenotypes, one can analyze the correlation of genetic mutations and phenotypes at the cellular level, which can be achieved by imaging and RNAseq analysis at single cell resolution (Zhang et al., 2021). Designing specific gRNAs and controlling Cas9 expression should minimize the off-targets and increase the specificity of CRSIPR/Cas9-mediated gene targeting. The heterogeneity in genetic backgrounds of large animals may lead to more variability in behaviors such that sufficient numbers of large animals are required to make a sound conclusion. For example, cognitive impairment is a common non-motor symptom in patients of NDs. Most of the NDs exhibit different extents of impaired cognitive functions that can be measured using Wisconsin general test apparatus (WGTA) (Zhou et al., 2019). Anxiety-associated behaviors or motor deficits can be examined and monitored by the locomotion activity assay (Liu et al., 2016). To assess sleep disturbance in large animal models, daily home-cage videos should be recorded at specific time points. Thus, the robust and automated systems for tracking and analyzing behavior will be important for accurately assessing species-specific normal behavior, disease phenotypes, and data reproducibility.

Alternatively, genetic modification can be performed in somatic cells to mirror neuropathology in the specific brain regions in large animals. The direct administration of AAV expressing gRNA/Cas9 or transgenes into specific brain regions of large animals at multiple developmental stages or different ages could more rapidly recapitulate brain region-dependent or age-related neuropathology. Treatment of gene-targeted animals with environmental stress or chemicals that induce oxidative stress could also facilitate disease progression, as oxidative stress has been implicated in the progression of a number of NDs. Moreover, the continued improvement of viral vectors that can readily penetrate the blood–brain barrier may greatly facilitate the delivery of the gRNA/Cas9 system or transgenes in the brains of large animals (Chan et al., 2017; Goertsen et al., 2021).

## Conclusions and perspectives

This review updates the large animal models of neurodegenerative diseases, including HD, PD and ALS. Although investigation of large animal models led to new discoveries of important pathological events or mechanism that have not been found in small animals, there are challenges and limitations that are largely stemmed from the costly and time-consuming investigation. However, the important information gained from the large animal models would be highly valuable for understanding the pathogenic mechanisms and identifying new therapeutic targets. Given the lack of obvious neurodegeneration phenotypes in most of genetically modified rodent models, the demand for establishing large animal models to study neurodegenerative diseases is well-appreciated, and the publications of large animal models with gene editing are steadily increased recently. It is expected that more of large animal models would be embraced as the preferred model for human diseases and translational medicine research. Moreover, the important information gained from large animal models would be highly valuable for generating more humanized mouse models. Specifically, important differences in pathological phenotypes between small and large animals would guide us to create humanized mouse models that can more closely emulate the important pathological features seen in large animal models and patients.

## Declarations

This work was supported by The National Natural Science Foundation of China (81830032, 32070534, 31872779, 82071421); Key Field Research and Development Program of Guangdong province (2018B030337001), Department of Science and Technology of Guangdong Province (2021ZT09Y007; 2020B121201006), Guangzhou Key Research Program on Brain Science (202007030008), The National Key Research and Development Program of China Stem Cell and Translational Research (2017YFA0105102).

WY and PY wrote the manuscript. X-J L and SL edited the manuscript. All discussed and approved the overall structure of the review.

### Abbreviations

AAV, adeno-associated viruses; AD, Alzheimer’s disease; ALS, amyotrophic lateral sclerosis; CMV, cytomegalovirus; CRISPR, clustered regularly interspaced short palindromic repeats; FTLD, fronto-temporal lobar degeneration; FUS, fused in sarcoma; HD, Huntington’s disease; HTT, huntingtin; NDs, neurodegenerative diseases; PD, Parkinson’s diseases; polyQ, polyglutamine; PRKN, parkin RBR E3 ubiquitin protein ligase ; SCNT, somatic cell nuclear transfer; sgRNA, single guide RNA; SNs, substantia nigras; SNCA, α-synuclein; SOD1, superoxide dismutase 1; SQSTM1, Sequestosome-1; SVZ, subventricular zone; TDP-43, TAR DNA-binding protein 43.

## References

[CR1] Akundi RS, Huang Z, Eason J, Pandya JD, Zhi L, Cass WA, Sullivan PG, Bueler H (2011). Increased mitochondrial calcium sensitivity and abnormal expression of innate immunity genes precede dopaminergic defects in Pink1-deficient mice. PLoS ONE.

[CR2] Arai T, Hasegawa M, Akiyama H, Ikeda K, Nonaka T, Mori H, Mann D, Tsuchiya K, Yoshida M, Hashizume Y (2006). TDP-43 is a component of ubiquitin-positive tau-negative inclusions in frontotemporal lobar degeneration and amyotrophic lateral sclerosis. Biochem Biophys Res Commun.

[CR3] Bai D, Yin P, Zhang Y, Sun F, Chen L, Lin L, Yan S, Li S, Li X-J (2021). Lack of association of somatic CAG repeat expansion with striatal neurodegeneration in HD knock-in animal models. Hum Mol Genet.

[CR4] Bakken TE, Miller JA, Ding S-L, Sunkin SM, Smith KA, Ng L, Szafer A, Dalley RA, Royall JJ, Lemon T (2016). A comprehensive transcriptional map of primate brain development. Nature.

[CR5] Bates GP, Dorsey R, Gusella JF, Hayden MR, Kay C, Leavitt BR, Nance M, Ross CA, Scahill RI, Wetzel R (2015). Huntington disease. Nat Rev Dis Prim.

[CR6] Baxa M, Hruska-Plochan M, Juhas S, Vodicka P, Pavlok A, Juhasova J, Miyanohara A, Nejime T, Klima J, Macakova M (2013). A transgenic minipig model of Huntington’s disease. J Huntingtons Dis.

[CR7] Bernard A, Lubbers LS, Tanis KQ, Luo R, Podtelezhnikov AA, Finney EM, McWhorter MME, Serikawa K, Lemon T, Morgan R (2012). Transcriptional architecture of the primate neocortex. Neuron.

[CR8] Chan KY, Jang MJ, Yoo BB, Greenbaum A, Ravi N, Wu W-L, Sánchez-Guardado L, Lois C, Mazmanian SK, Deverman BE (2017). Engineered AAVs for efficient noninvasive gene delivery to the central and peripheral nervous systems. Nat Neurosci.

[CR9] Chen B, Niu Y, Wang H, Wang K, Yang H, Li W (2020). Recent advances in CRISPR research. Protein Cell.

[CR10] Chen Z-Z, Wang J-Y, Kang Y, Yang Q-Y, Gu X-Y, Zhi D-L, Yan L, Long C-Z, Shen B, Niu Y-Y (2021). PINK1 gene mutation by pair truncated sgRNA/Cas9-D10A in cynomolgus monkeys. Zool Res.

[CR11] Chen-Plotkin AS, Lee VM-Y, Trojanowski JQ (2010). TAR DNA-binding protein 43 in neurodegenerative disease. Nat Rev Neurol.

[CR12] Chieppa MN, Perota A, Corona C, Grindatto A, Lagutina I, Vallino Costassa E, Lazzari G, Colleoni S, Duchi R, Lucchini F (2014). Modeling amyotrophic lateral sclerosis in hSOD1 transgenic swine. Neurodegener Dis.

[CR13] Corti O, Lesage S, Brice A (2011). What genetics tells us about the causes and mechanisms of Parkinson’s disease. Physiol Rev.

[CR14] Crook ZR, Housman D (2011). Huntington’s disease: can mice lead the way to treatment?. Neuron.

[CR15] Cummins N, Gotz J (2018). Shedding light on mitophagy in neurons: what is the evidence for PINK1/Parkin mitophagy in vivo?. Cell Mol Life Sci.

[CR16] Damier P, Hirsch EC, Agid Y, Graybiel AM (1999). The substantia nigra of the human brain. II. Patterns of loss of dopamine-containing neurons in Parkinson’s disease. Brain.

[CR17] Darmanis S, Sloan SA, Zhang Y, Enge M, Caneda C, Shuer LM, Hayden Gephart MG, Barres BA, Quake SR (2015). A survey of human brain transcriptome diversity at the single cell level. Proc Natl Acad Sci U S A.

[CR18] Davies SW, Turmaine M, Cozens BA, DiFiglia M, Sharp AH, Ross CA, Scherzinger E, Wanker EE, Mangiarini L, Bates GP (1997). Formation of neuronal intranuclear inclusions underlies the neurological dysfunction in mice transgenic for the HD mutation. Cell.

[CR19] Dawson TM, Golde TE, Lagier-Tourenne C (2018). Animal models of neurodegenerative diseases. Nat Neurosci.

[CR20] Defelipe J (2011). The evolution of the brain, the human nature of cortical circuits, and intellectual creativity. Front Neuroanat.

[CR21] Deng HX, Siddique T (2000). Transgenic mouse models and human neurodegenerative disorders. Arch Neurol.

[CR22] Deng H, Wang P, Jankovic J (2018). The genetics of Parkinson disease. Ageing Res Rev.

[CR23] Dugger BN, Perl DP, Carlson GA (2017). Neurodegenerative disease transmission and transgenesis in mice. Cold Spring Harb Perspect Biol.

[CR24] Farshim PP, Bates GP (2018). Mouse models of Huntington’s disease. Methods Mol Biol.

[CR25] Ferraiuolo L, Kirby J, Grierson AJ, Sendtner M, Shaw PJ (2011). Molecular pathways of motor neuron injury in amyotrophic lateral sclerosis. Nat Rev Neurol.

[CR26] Forsberg K, Andersen PM, Marklund SL, Brännström T (2011). Glial nuclear aggregates of superoxide dismutase-1 are regularly present in patients with amyotrophic lateral sclerosis. Acta Neuropathol.

[CR27] Gao P, Postiglione MP, Krieger TG, Hernandez L, Wang C, Han Z, Streicher C, Papusheva E, Insolera R, Chugh K (2014). Deterministic progenitor behavior and unitary production of neurons in the neocortex. Cell.

[CR28] Gautier CA, Kitada T, Shen J (2008). Loss of PINK1 causes mitochondrial functional defects and increased sensitivity to oxidative stress. Proc Natl Acad Sci U S A.

[CR29] Geschwind DH, Rakic P (2013). Cortical evolution: judge the brain by its cover. Neuron.

[CR30] Giasson BI, Duda JE, Quinn SM, Zhang B, Trojanowski JQ, Lee VM-Y (2002). Neuronal alpha-synucleinopathy with severe movement disorder in mice expressing A53T human alpha-synuclein. Neuron.

[CR31] Gispert S, Ricciardi F, Kurz A, Azizov M, Hoepken H-H, Becker D, Voos W, Leuner K, Muller WE, Kudin AP (2009). Parkinson phenotype in aged PINK1-deficient mice is accompanied by progressive mitochondrial dysfunction in absence of neurodegeneration. PLoS ONE.

[CR32] Goertsen D, Flytzanis NC, Goeden N, Chuapoco MR, Cummins A, Chen Y, Fan Y, Zhang Q, Sharma J, Duan Y (2021). AAV capsid variants with brain-wide transgene expression and decreased liver targeting after intravenous delivery in mouse and marmoset. Nat Neurosci.

[CR33] Goldberg MS, Fleming SM, Palacino JJ, Cepeda C, Lam HA, Bhatnagar A, Meloni EG, Wu N, Ackerson LC, Klapstein GJ (2003). Parkin-deficient mice exhibit nigrostriatal deficits but not loss of dopaminergic neurons. J Biol Chem.

[CR34] Grad LI, Rouleau GA, Ravits J, Cashman NR (2017). Clinical spectrum of amyotrophic lateral sclerosis (ALS). Cold Spring Harb Perspect Med.

[CR35] Hauschild J, Petersen B, Santiago Y, Queisser A-L, Carnwath JW, Lucas-Hahn A, Zhang L, Meng X, Gregory PD, Schwinzer R (2011). Efficient generation of a biallelic knockout in pigs using zinc-finger nucleases. Proc Natl Acad Sci USA.

[CR36] Hawrylycz M, Miller JA, Menon V, Feng D, Dolbeare T, Guillozet-Bongaarts AL, Jegga AG, Aronow BJ, Lee C-K, Bernard A (2015). Canonical genetic signatures of the adult human brain. Nat Neurosci.

[CR37] Herculano-Houzel S (2012). The remarkable, yet not extraordinary, human brain as a scaled-up primate brain and its associated cost. Proc Natl Acad Sci USA.

[CR38] Huang C, Tong J, Bi F, Zhou H, Xia X-G (2012). Mutant TDP-43 in motor neurons promotes the onset and progression of ALS in rats. J Clin Invest.

[CR39] Incontro S, Asensio CS, Edwards RH, Nicoll RA (2014). Efficient, complete deletion of synaptic proteins using CRISPR. Neuron.

[CR40] Ip CW, Klaus L-C, Karikari AA, Visanji NP, Brotchie JM, Lang AE, Volkmann J, Koprich JB (2017). AAV1/2-induced overexpression of A53T-α-synuclein in the substantia nigra results in degeneration of the nigrostriatal system with Lewy-like pathology and motor impairment: a new mouse model for Parkinson’s disease. Acta Neuropathol Commun.

[CR41] Izpisua Belmonte JC, Callaway EM, Caddick SJ, Churchland P, Feng G, Homanics GE, Lee K-F, Leopold DA, Miller CT, Mitchell JF (2015). Brains, genes, and primates. Neuron.

[CR42] Jacobsen JC, Bawden CS, Rudiger SR, McLaughlan CJ, Reid SJ, Waldvogel HJ, MacDonald ME, Gusella JF, Walker SK, Kelly JM (2010). An ovine transgenic Huntington’s disease model. Hum Mol Genet.

[CR43] Kakita A, Oyanagi K, Nagai H, Takahashi H (1997). Eosinophilic intranuclear inclusions in the hippocampal pyramidal neurons of a patient with amyotrophic lateral sclerosis. Acta Neuropathol.

[CR44] Kitada T, Pisani A, Porter DR, Yamaguchi H, Tscherter A, Martella G, Bonsi P, Zhang C, Pothos EN, Shen J (2007). Impaired dopamine release and synaptic plasticity in the striatum of PINK1-deficient mice. Proc Natl Acad Sci USA.

[CR45] Kitada T, Tong Y, Gautier CA, Shen J (2009). Absence of nigral degeneration in aged parkin/DJ-1/PINK1 triple knockout mice. J Neurochem.

[CR46] Lagier-Tourenne C, Cleveland DW (2009). Rethinking ALS: the FUS about TDP-43. Cell.

[CR47] Lanoiselée H-M, Nicolas G, Wallon D, Rovelet-Lecrux A, Lacour M, Rousseau S, Richard A-C, Pasquier F, Rollin-Sillaire A, Martinaud O (2017). APP, PSEN1, and PSEN2 mutations in early-onset Alzheimer disease: a genetic screening study of familial and sporadic cases. PLoS Med.

[CR48] Lee MK, Stirling W, Xu Y, Xu X, Qui D, Mandir AS, Dawson TM, Copeland NG, Jenkins NA, Price DL (2002). Human alpha-synuclein-harboring familial Parkinson’s disease-linked Ala-53 –> Thr mutation causes neurodegenerative disease with alpha-synuclein aggregation in transgenic mice. Proc Natl Acad Sci USA.

[CR49] Levine MS, Cepeda C, Hickey MA, Fleming SM, Chesselet M-F (2004). Genetic mouse models of Huntington’s and Parkinson’s diseases: illuminating but imperfect. Trends Neurosci.

[CR50] Li H, Wu S, Ma X, Li X, Cheng T, Chen Z, Wu J, Lv L, Li L, Xu L (2021). Co-editing PINK1 and DJ-1 genes via adeno-associated virus-delivered CRISPR/Cas9 system in adult monkey brain elicits classical Parkinsonian phenotype. Neurosci Bull.

[CR51] Liu Z, Li X, Zhang JT, Cai YJ, Cheng TL, Cheng C, Wang Y, Zhang CC, Nie YH, Chen ZF (2016). Autism-like behaviours and germline transmission in transgenic monkeys overexpressing MeCP2. Nature.

[CR52] Liu Z, Cai Y, Wang Y, Nie Y, Zhang C, Xu Y, Zhang X, Lu Y, Wang Z, Poo M (2018). Cloning of macaque monkeys by somatic cell nuclear transfer. Cell.

[CR53] Lunney JK, Van Goor A, Walker KE, Hailstock T, Franklin J, Dai C (2021). Importance of the pig as a human biomedical model. Sci Transl Med.

[CR54] McInerney-Leo A, Hadley DW, Gwinn-Hardy K, Hardy J (2005). Genetic testing in Parkinson’s disease. Mov Disord.

[CR55] Mitchell JC, Constable R, So E, Vance C, Scotter E, Glover L, Hortobagyi T, Arnold ES, Ling S-C, McAlonis M (2015). Wild type human TDP-43 potentiates ALS-linked mutant TDP-43 driven progressive motor and cortical neuron degeneration with pathological features of ALS. Acta Neuropathol Commun.

[CR56] Molyneaux BJ, Goff LA, Brettler AC, Chen H-H, Hrvatin S, Rinn JL, Arlotta P (2015). DeCoN: genome-wide analysis of in vivo transcriptional dynamics during pyramidal neuron fate selection in neocortex. Neuron.

[CR57] Neumann M, Sampathu DM, Kwong LK, Truax AC, Micsenyi MC, Chou TT, Bruce J, Schuck T, Grossman M, Clark CM (2006). Ubiquitinated TDP-43 in frontotemporal lobar degeneration and amyotrophic lateral sclerosis. Science.

[CR58] Niu Y, Guo X, Chen Y, Wang C-E, Gao J, Yang W, Kang Y, Si W, Wang H, Yang S-H (2015). Early Parkinson’s disease symptoms in alpha-synuclein transgenic monkeys. Hum Mol Genet.

[CR59] Orr HT, Zoghbi HY (2007). Trinucleotide repeat disorders. Annu Rev Neurosci.

[CR60] Otani T, Marchetto MC, Gage FH, Simons BD, Livesey FJ (2016). 2D and 3D stem cell models of primate cortical development identify species-specific differences in progenitor behavior contributing to brain size. Cell Stem Cell.

[CR61] Perez FA, Palmiter RD (2005). Parkin-deficient mice are not a robust model of parkinsonism. Proc Natl Acad Sci USA.

[CR62] Philips T, Rothstein JD (2015). Rodent models of amyotrophic lateral sclerosis. Curr Protoc Pharmacol.

[CR63] Pickrell AM, Youle RJ (2015). The roles of PINK1, parkin, and mitochondrial fidelity in Parkinson’s disease. Neuron.

[CR64] Polymenidou M, Lagier-Tourenne C, Hutt KR, Huelga SC, Moran J, Liang TY, Ling S-C, Sun E, Wancewicz E, Mazur C (2011). Long pre-mRNA depletion and RNA missplicing contribute to neuronal vulnerability from loss of TDP-43. Nat Neurosci.

[CR65] Rash BG, Duque A, Morozov YM, Arellano JI, Micali N, Rakic P (2019). Gliogenesis in the outer subventricular zone promotes enlargement and gyrification of the primate cerebrum. Proc Natl Acad Sci USA.

[CR66] Reid SJ, Patassini S, Handley RR, Rudiger SR, McLaughlan CJ, Osmand A, Jacobsen JC, Morton AJ, Weiss A, Waldvogel HJ (2013). Further molecular characterisation of the OVT73 transgenic sheep model of Huntington’s disease identifies cortical aggregates. J Huntingtons Dis.

[CR67] Rubinsztein DC (2006). The roles of intracellular protein-degradation pathways in neurodegeneration. Nature.

[CR68] Ryu J, Prather RS, Lee K (2018). Use of gene-editing technology to introduce targeted modifications in pigs. J Anim Sci Biotechnol.

[CR117] Schuldenzucker Verena, Schubert Robin, Muratori Lisa M., Freisfeld Frauke, Rieke Lorena, Matheis Tamara, Schramke Sarah, Motlik Jan, Kemper Nicole, Radespiel Ute (2017). Behavioral testing of minipigs transgenic for the Huntington gene—A three-year observational study. PLOS ONE.

[CR69] Seilhean D, Takahashi J, El Hachimi KH, Fujigasaki H, Lebre A-S, Biancalana V, Dürr A, Salachas F, Hogenhuis J, de Thé H (2004). Amyotrophic lateral sclerosis with neuronal intranuclear protein inclusions. Acta Neuropathol.

[CR70] Shan X, Chiang P-M, Price DL, Wong PC (2010). Altered distributions of Gemini of coiled bodies and mitochondria in motor neurons of TDP-43 transgenic mice. Proc Natl Acad Sci USA.

[CR71] Smart IHM, Dehay C, Giroud P, Berland M, Kennedy H (2002). Unique morphological features of the proliferative zones and postmitotic compartments of the neural epithelium giving rise to striate and extrastriate cortex in the monkey. Cereb Cortex.

[CR72] Soto C, Pritzkow S (2018). Protein misfolding, aggregation, and conformational strains in neurodegenerative diseases. Nat Neurosci.

[CR73] Sousa AMM, Zhu Y, Raghanti MA, Kitchen RR, Onorati M, Tebbenkamp ATN, Stutz B, Meyer KA, Li M, Kawasawa YI (2017). Molecular and cellular reorganization of neural circuits in the human lineage. Science.

[CR74] Sun Z, Ye J, Yuan J (2021). PINK1 mediates neuronal survival in monkey. Protein Cell.

[CR75] Swami M, Hendricks AE, Gillis T, Massood T, Mysore J, Myers RH, Wheeler VC (2009). Somatic expansion of the Huntington’s disease CAG repeat in the brain is associated with an earlier age of disease onset. Hum Mol Genet.

[CR76] Swiech L, Heidenreich M, Banerjee A, Habib N, Li Y, Trombetta J, Sur M, Zhang F (2015). In vivo interrogation of gene function in the mammalian brain using CRISPR-Cas9. Nat Biotechnol.

[CR77] Tabrizi SJ, Flower MD, Ross CA, Wild EJ (2020). Huntington disease: new insights into molecular pathogenesis and therapeutic opportunities. Nat Rev Neurol.

[CR78] Turner MR, Hardiman O, Benatar M, Brooks BR, Chio A, de Carvalho M, Ince PG, Lin C, Miller RG, Mitsumoto H (2013). Controversies and priorities in amyotrophic lateral sclerosis. Lancet Neurol.

[CR79] Uchida A, Sasaguri H, Kimura N, Tajiri M, Ohkubo T, Ono F, Sakaue F, Kanai K, Hirai T, Sano T (2012). Non-human primate model of amyotrophic lateral sclerosis with cytoplasmic mislocalization of TDP-43. Brain.

[CR80] Valente EM, Abou-Sleiman PM, Caputo V, Muqit MMK, Harvey K, Gispert S, Ali Z, Del Turco D, Bentivoglio AR, Healy DG (2004). Hereditary early-onset Parkinson’s disease caused by mutations in PINK1. Science.

[CR82] Wang G, Yang H, Yan S, Wang C-E, Liu X, Zhao B, Ouyang Z, Yin P, Liu Z, Zhao Y (2015). Cytoplasmic mislocalization of RNA splicing factors and aberrant neuronal gene splicing in TDP-43 transgenic pig brain. Mol Neurodegener.

[CR83] Wang X, Cao C, Huang J, Yao J, Hai T, Zheng Q, Wang X, Zhang H, Qin G, Cheng J (2016). One-step generation of triple gene-targeted pigs using CRISPR/Cas9 system. Sci Rep.

[CR84] Wang K, Jin Q, Ruan D, Yang Y, Liu Q, Wu H, Zhou Z, Ouyang Z, Liu Z, Zhao Y (2017). Cre-dependent Cas9-expressing pigs enable efficient in vivo genome editing. Genome Res.

[CR85] Wang F, Zhang W, Yang Q, Kang Y, Fan Y, Wei J, Liu Z, Dai S, Li H, Li Z (2020). Generation of a Hutchinson-Gilford progeria syndrome monkey model by base editing. Protein Cell.

[CR86] Wegorzewska I, Bell S, Cairns NJ, Miller TM, Baloh RH (2009). TDP-43 mutant transgenic mice develop features of ALS and frontotemporal lobar degeneration. Proc Natl Acad Sci USA.

[CR87] Weishaupt JH, Hyman T, Dikic I (2016). Common molecular pathways in amyotrophic lateral sclerosis and frontotemporal dementia. Trends Mol Med.

[CR88] Weiss AR, Liguore WA, Domire JS, Button D, McBride JL (2020). Intra-striatal AAV2.retro administration leads to extensive retrograde transport in the rhesus macaque brain: implications for disease modeling and therapeutic development. Sci Rep.

[CR89] Whitworth AJ, Pallanck LJ (2017). PINK1/Parkin mitophagy and neurodegeneration-what do we really know in vivo?. Curr Opin Genet Dev.

[CR90] Wils H, Kleinberger G, Janssens J, Pereson S, Joris G, Cuijt I, Smits V, Ceuterick-de Groote C, Van Broeckhoven C, Kumar-Singh S (2010). TDP-43 transgenic mice develop spastic paralysis and neuronal inclusions characteristic of ALS and frontotemporal lobar degeneration. Proc Natl Acad Sci U S A.

[CR91] Wyss-Coray T (2016). Ageing, neurodegeneration and brain rejuvenation. Nature.

[CR92] Xie J, Ge W, Li N, Liu Q, Chen F, Yang X, Huang X, Ouyang Z, Zhang Q, Zhao Y (2019). Efficient base editing for multiple genes and loci in pigs using base editors. Nat Commun.

[CR93] Xiong H, Wang D, Chen L, Choo YS, Ma H, Tang C, Xia K, Jiang W, Ronai Z, Zhuang X (2009). Parkin, PINK1, and DJ-1 form a ubiquitin E3 ligase complex promoting unfolded protein degradation. J Clin Invest.

[CR94] Yan S, Wang C-E, Wei W, Gaertig MA, Lai L, Li S, Li X-J (2014). TDP-43 causes differential pathology in neuronal versus glial cells in the mouse brain. Hum Mol Genet.

[CR95] Yan S, Tu Z, Liu Z, Fan N, Yang H, Yang S, Yang W, Zhao Y, Ouyang Z, Lai C (2018). A Huntingtin knockin pig model recapitulates features of selective neurodegeneration in Huntington’s disease. Cell.

[CR96] Yang S-H, Cheng P-H, Banta H, Piotrowska-Nitsche K, Yang J-J, Cheng ECH, Snyder B, Larkin K, Liu J, Orkin J (2008). Towards a transgenic model of Huntington’s disease in a non-human primate. Nature.

[CR97] Yang D, Wang C-E, Zhao B, Li W, Ouyang Z, Liu Z, Yang H, Fan P, O’Neill A, Gu W (2010). Expression of Huntington’s disease protein results in apoptotic neurons in the brains of cloned transgenic pigs. Hum Mol Genet.

[CR98] Yang H, Wang G, Sun H, Shu R, Liu T, Wang C-E, Liu Z, Zhao Y, Zhao B, Ouyang Z (2014). Species-dependent neuropathology in transgenic SOD1 pigs. Cell Res.

[CR99] Yang W, Wang G, Wang C-E, Guo X, Yin P, Gao J, Tu Z, Wang Z, Wu J, Hu X (2015). Mutant alpha-synuclein causes age-dependent neuropathology in monkey brain. J Neurosci.

[CR100] Yang W, Li S, Li X-J (2019). A CRISPR monkey model unravels a unique function of PINK1 in primate brains. Mol Neurodegener.

[CR101] Yang W, Liu Y, Tu Z, Xiao C, Yan S, Ma X, Guo X, Chen X, Yin P, Yang Z (2019). CRISPR/Cas9-mediated PINK1 deletion leads to neurodegeneration in rhesus monkeys. Cell Res.

[CR102] Yang W, Chen X, Li S, Li X-J (2021). Genetically modified large animal models for investigating neurodegenerative diseases. Cell Biosci.

[CR103] Yang W, Guo X, Tu Z, Chen X, Han R, Liu Y, Yan S, Wang Q, Wang Z, Zhao X (2021). PINK1 kinase dysfunction triggers neurodegeneration in the primate brain without impacting mitochondrial homeostasis. Protein Cell.

[CR104] Yin P, Guo X, Yang W, Yan S, Yang S, Zhao T, Sun Q, Liu Y, Li S, Li X-J (2019). Caspase-4 mediates cytoplasmic accumulation of TDP-43 in the primate brains. Acta Neuropathol.

[CR105] Yin P, Bai D, Deng F, Zhang C, Jia Q, Zhu L, Chen L, Li B, Guo X, Ye J (2021). SQSTM1-mediated clearance of cytoplasmic mutant TARDBP/TDP-43 in the monkey brain. Autophagy.

[CR106] Zeisel A, Muñoz-Manchado AB, Codeluppi S, Lönnerberg P, La Manno G, Juréus A, Marques S, Munguba H, He L, Betsholtz C (2015). Brain structure. Cell types in the mouse cortex and hippocampus revealed by single-cell RNA-seq. Science.

[CR107] Zeng H, Shen EH, Hohmann JG, Oh SW, Bernard A, Royall JJ, Glattfelder KJ, Sunkin SM, Morris JA, Guillozet-Bongaarts AL (2012). Large-scale cellular-resolution gene profiling in human neocortex reveals species-specific molecular signatures. Cell.

[CR108] Zhang W, Wan H, Feng G, Qu J, Wang J, Jing Y, Ren R, Liu Z, Zhang L, Chen Z (2018). SIRT6 deficiency results in developmental retardation in cynomolgus monkeys. Nature.

[CR109] Zhang H, Li J, Ren J, Sun S, Ma S, Zhang W, Yu Y, Cai Y, Yan K, Li W (2021). Single-nucleus transcriptomic landscape of primate hippocampal aging. Protein Cell.

[CR110] Zhao H, Tu Z, Xu H, Yan S, Yan H, Zheng Y, Yang W, Zheng J, Li Z, Tian R (2017). Altered neurogenesis and disrupted expression of synaptic proteins in prefrontal cortex of SHANK3-deficient non-human primate. Cell Res.

[CR111] Zhao J, Lai L, Ji W, Zhou Q (2019). Genome editing in large animals: current status and future prospects. Natl Sci Rev.

[CR112] Zhou H, Falkenburger BH, Schulz JB, Tieu K, Xu Z, Xia XG (2007). Silencing of the Pink1 gene expression by conditional RNAi does not induce dopaminergic neuron death in mice. Int J Biol Sci.

[CR113] Zhou X, Xin J, Fan N, Zou Q, Huang J, Ouyang Z, Zhao Y, Zhao B, Liu Z, Lai S (2015). Generation of CRISPR/Cas9-mediated gene-targeted pigs via somatic cell nuclear transfer. Cell Mol Life Sci.

[CR114] Zhou Y, Sharma J, Ke Q, Landman R, Yuan J, Chen H, Hayden DS, Fisher JW, Jiang M, Menegas W (2019). Atypical behaviour and connectivity in SHANK3-mutant macaques. Nature.

[CR115] Zhu Y, Sousa AMM, Gao T, Skarica M, Li M, Santpere G, Esteller-Cucala P, Juan D, Ferrández-Peral L, Gulden FO (2018). Spatiotemporal transcriptomic divergence across human and macaque brain development. Science.

[CR116] Zoghbi HY, Botas J (2002). Mouse and fly models of neurodegeneration. Trends Genet.

